# Early retinal neurovascular findings in post-transplant diabetes mellitus patients without clinical signs of diabetic retinopathy

**DOI:** 10.1186/s40942-023-00487-4

**Published:** 2023-08-23

**Authors:** Anne Elise Cruz do Carmo Chaves, Thizá Massaia Londero, Monica Oliveira da Silva, Fábio Lavinsky, Cristiane Bauermann Leitão, Andrea Carla Bauer, Daniel Lavinsky

**Affiliations:** 1 Post-Graduate Program in Medical Sciences, Endocrinology, Universidade Federal do Rio Grande do Sul (UFRGS), Porto Alegre, 97105-900 Rio Grande do Sul Brazil; 2 Clinical Medicine Department, Universidade Federal de Santa Maria, Santa Maria, Rio Grande do Sul Brazil; 3 Endocrinology Division, Hospital de Clínicas de Porto Alegre (HCPA), Porto Alegre, 90035-903 Rio Grande do Sul Brazil; 4 Nephrology Division, Hospital de Clínicas de Porto Alegre (HCPA), Port Alegre, 90035-903 Rio Grande do Sul Brazil; 5 Ophthalmology Division, Hospital de Clínicas de Porto Alegre, Porto Alegre, 90035-903 Rio Grande do Sul Brazil

**Keywords:** Post-transplant diabetes mellitus, Diabetic retinopathy, Early retinal neurovascular findings

## Abstract

**Background:**

Post-transplant diabetes mellitus (PTDM) is a specific subtype of diabetes with an uncertain impact on mortality and morbidity in post-transplant patients. Diabetic retinopathy is the most common microvascular complication of diabetes mellitus, but the long-term clinical progression in PTDM is unknown. New technologies are being used to assess pre-clinical signs of retinal changes, such as swept-source optical coherence tomography (OCT) and OCT-angiography. The aim of this study was to detect pre-clinical structural and vascular changes in the retina using swept-source-OCT and OCT-angiography in patients with PTDM.

**Methods:**

In this retrospective cohort study, post-kidney transplant patients were divided into PTDM and non-PTDM (control) groups. Both eyes of eligible PTDM patients and controls were included in this study. Inner retinal layer thickness was measured with swept-source-OCT. Retinal capillary density and the foveal avascular zone were measured with OCT-angiography.

**Results:**

In the PTDM group, reduced thickness was found in the inferior ganglion cell layer plus inner plexiform layer (95% CI -8.76 to -0.68; p = 0.022) and the temporal inferior segment (95% CI -10.23 to -0.76; p = 0.024) of the inner retina, as well as in the retinal nerve fiber layer in the temporal (95% CI -34.78 to -9.28 p = 0.001) and temporal inferior segments (95% CI -33.26 to -5.03 p = 0.008). No significant differences were found in the vascular capillary plexus between groups at all depths, segments, or foveal avascular zone (p = 0.088).

**Conclusions:**

According to OCT-angiography, PTDM patients had reduced inner neurosensory retinal layers but no significant change in vascular density, which suggests that early neuroretinal degeneration might occur prior to vascular changes secondary to PTDM. Prospective studies could help elucidate the clinical course of retinal neuropathy and microvascular pathology in PTDM and provide a better understanding of PTDM complications.

**Supplementary Information:**

The online version contains supplementary material available at 10.1186/s40942-023-00487-4.

## Introduction

Diabetes mellitus (DM) is a group of heterogeneous disorders involving increased blood glucose concentrations. Once hyperglycemia occurs, patients with all forms of diabetes are at risk of developing the same chronic complications, although the rates of progression may differ [1, 2]. The International Diabetes Federation estimated the global population with diabetes mellitus to be 463,000,000 in 2019 and has projected it to be 700,000,000 by 2045 [3].

Post-transplant DM (PTDM), a subtype of diabetes defined in 2014, occurs in previously nondiabetic people after a solid organ or hematopoietic transplantation [4]. The pathogenesis of PTDM is related to the use of corticosteroids and calcineurin inhibitors in the post-transplant period and shares some of the same risk factors as type 2 DM, such as obesity and older age [5]. PTDM is associated with higher rates of postoperative infection and cardiovascular events, which are the leading causes of death in kidney transplant recipients [6].

Diabetic retinopathy (DR) is the most common microvascular complication of DM and is the leading cause of vision loss in in the adult working population [7, 8]. DR is typically classified according to a severity scale based on fundus examination findings (microaneurysms, hemorrhages, and vascular changes or neovascularization) [9]. The long-term clinical progression in PTDM is unknown. Our group in a previous cohort of post-transplant patients with PTDM evaluate the clinical course of diabetic microvascular complications, assessing diabetic kidney disease (DKD), polyneuropathy, autonomic cardiovascular neuropathy as well as diabetic retinopathy. No clinical signs of DR was found in retinography performed 5 years after diabetes diagnosis, but at SS-OCT examination a decrease in the thickness of inner retinal layers was observed in kidney transplant recipients with PTDM in comparison to those without PTDM [10].

Many studies on DR have found pre-clinical abnormalities in retinal layer analyses using swept-source optical coherence tomography (SS-OCT) and optical coherence tomography angiography (OCTA) [11–14]. OCTA is a non-invasive imaging technique that generates volumetric angiography images in a few seconds. It provides a highly detailed reconstruction of the retinal vasculature, which allows for accurate delineation of the foveal avascular zone (FAZ) in diabetic eyes [15, 16] .

The aim of this study was to analyze specific retinal microvascular findings and detect early structural and vascular density changes in the retina, FAZ, and inner retinal layers using SS-OCT and OCTA in patients who have been diagnosed with PTDM for at least 5 years.

## Methods

A retrospective cohort study was conducted with all patients who received kidney transplant recipients at the Hospital de Clínicas de Porto Alegre (Porto Alegre, RS, Brazil) between January 2000 and December 2011. The Hospital’s ethics committee approved this study. All participants provided written informed consent, and this study complies with the principles of the Declaration of Helsinki and good clinical practice.

The inclusion criteria were at least 5 years since PTDM diagnosis, age > 18 years, and no history of DM prior to transplantation. PTDM was diagnosed according to the American Diabetes Association criteria, [17] and episodes of transient hyperglycemia related to high doses of corticosteroid and/or tacrolimus early after transplantation were not considered PTDM. The exclusion criteria were kidney graft loss, death, loss to follow-up, history of any significant ocular disease, previous diagnosis of glaucoma, or media opacities.

The control group consisted of kidney transplant patients during the same period with no diagnosis of PTDM. Exclusion criteria were kidney graft loss, death, or loss to follow-up, in addition to any ophthalmological condition that could interfere with the imaging assessment.

Both eyes of eligible PTDM patients and controls were included in this study. All patients underwent SS-OCT and OCTA with a DRI OCT Triton system (Topcon, Tokyo, Japan). Demographic data were also collected.

### SS-OCT and OCTA Imaging

The images were analyzed in Topcon IMAGEnet 6 v1.22. The imaging protocol included wide macula, line macula, and simultaneous color fundus of the posterior pole. All patients were evaluated with SS-OCT by an experienced ophthalmologist, and all OCT scans were analyzed by a blinded investigator for quality, being excluded if they showed signs of low quality or a significant artifact.

OCTA scans were acquired using the DRI OCT Triton system based on the Topcon OCT angiography ratio analysis algorithm. The imaging protocol was a 6 × 6 mm Early Treatment DR Study grid overlay centered on the fovea. Boundary layer segmentations were defined as superficial capillary plexus from 2.6 μm below internal limiting membrane to 15.6 μm below the junction between inner plexiform layer and the inner nuclear layer, deep capillary plexus from 15.6 to 70.2 μm below this junction, and choriocapillaris from Bruch’s membrane (0-µm offset) to 10.4 μm below Bruch’s membrane. OCTA software determines the proportion of bright to dark pixels to derive a measure of vascular density. Vascular density is derived from the percentage of the area occupied by bright pixels in a segmented area. The FAZ (µm^2^) was measured through manual delineation in the deep plexus using SS-OCT software. Retinal thickness and vascular density measurements were obtained from an Early Treatment DR Study grid centered on the fovea (diameters: center 1 mm, inner circle 3 mm, outer circle 6 mm). Automated segmentation of the retina was performed in 9 Early Treatment DR Study areas, in addition to average thickness, center thickness, and total volume. The ganglion cell layer plus inner plexiform layer (GCL+), i.e., from the nerve fiber layer/ganglion cell layer to the inner plexiform layer/inner nuclear layer, and the retinal nerve fiber layer (RNFL) segmentation, was determined in 6 standard areas: superior, nasal superior, temporal superior, inferior, temporal inferior, nasal inferior, and total volume.

### Statistics

Demographic and descriptive data were expressed as mean (SD). Continuous variables were presented as mean (SD), while categorical variables were presented as percentages. Differences between groups were assessed with generalized estimating equations and were adjusted for both eyes, excluding missing values. Statistical analysis was performed using IBM SPSS Statistics 28.0 (IBM, Armonk, NY, USA).

## Results

A total of 895 patients received a kidney transplant at the Hospital between January 2000 and December 2011, of whom 135 (15%) developed PTDM over 144.5 months of follow-up. Of these, 64 had been diagnosed with PTDM for > 5 years and were considered eligible for this study. After applying the exclusion criteria, 35 patients were included in PTDM group, and 48 patients without PTDM were included in control group. Most participants were male (n = 54, 65.06%), with a mean age was 53.78 years. The demographic and clinical findings are shown in Table 1.

The SS-OCT analysis consisted of 162 eyes (83 patients), 68 in the PTDM group and 94 in the control group. Age-adjusted inner retinal layer measurement showed reduced thickness in the PTDM GCL+ (95% CI -8.76 to -0.68; p = 0.022) and in the temporal inferior segment (95% CI -10.23 to -0.76; p = 0.024), as well as RNFL reduction in the temporal superior (95% CI -34.78 to -9.28; p = 0.001) and temporal inferior segments (95% CI -33.26 to -5.03; p = 0.008) (Table 2).

OCTA density was measured in 139 eyes, 61 in the PTDM group and 78 in the control group. After image quality assessment, 134 eyes remained eligible (73 participants): 58 eyes in PTDM group and 76 in control group. No significant difference was found between groups for the vascular capillary plexus at all depths and segments (superficial central density 95% CI -5.08 to 0.082; p = 0.058; deep central 95% CI -4.67 to 0.56; p = 0.123; choriocapillaris central 95% CI -3.26 to 2.75; p = 0.867). The mean capillary density values of the superficial capillary plexus, deep plexus, and choriocapillaris are shown in Table 3. No significant enlargement was observed in PTDM FAZ compared to controls (95% CI, -9.12 to 131.76; p = 0.088) (Table 3).

**Table 1:** Demographic and clinical characteristics of PTDM patients and non-PTDM controls

	PTDM (35)	Controls (48)	p-value
Age (years)	58.31	51.04	0.001
Sex – Male (%)	57.14	70.83	0.495
HbA1c (mean)	7.54	5.42	0.001
Caucasian (%)	80	78.4	1.00
Parental DM (%)	40	43.2	0.931

DM: diabetes mellitus

**Table 2:** Mean retinal layer thickness (µm) in PTDM patients and non-PTDM controls

Retinal Layer Thickness, ETDRS (µm)	PTDM (µm)	Control (µm)	95%CI	p-value
	(n = 68 eyes)	(n = 94 eyes)		
Retina				
Center retinal thickness	188.16	193.26	-19.72 to 9.54	0.495
Total volume	7.54	7.77	-0.5 to 0.4	0.089
Inner temporal	285.06	294.65	-22.54 to 3.38	0.147
Inner superior	295.86	304.80	-21.28 to 3.41	0.156
Inner nasal	292.38	303.96	-24.06 to 0.92	0.069
Inner inferior	293.55	302.71	-21.59 to 3.28	0.149
GCL+				
Total	65.65	69.61	-8.13 to 0.22	0.063
Temporal superior	66.49	69.18	-7.34 to 1.96	0.257
Superior	64.06	67.57	-7.87 to 0.86	0.115
Nasal superior	68.69	72.18	-8.23 to 1.25	0.149
Nasal inferior	67.53	70.91	-8.02 to 1.26	0.154
Inferior	61.64	66.36	-8.76 to -0.68	**0.022**
Temporal inferior	65.45	71.25	-10.23 to -0.76	**0.024**
RNFL				
Temporal	70.12	74.85	-11.51 to 2.04	0.171
Temporal superior	112.40	134.43	-34.78 to -9.28	**0.001**
Temporal inferior	120.33	139.47	-33.26 to -5.03	**0.008**
Nasal superior	111.98	117.35	-18.02 to 7.27	0.406
Nasal	83.31	88.04	-11.72 to 2.26	0.185
Nasal inferior	118.29	130.51	-24.77 to 0.34	0.057

ETDRS: Early Treatment Diabetic Retinopathy Study; GCL+: Ganglion cell layer plus inner plexiform layer; RNFL: retinal nerve fiber layer

**Table 3:** Mean capillary density in optical coherence tomography angiography in post-transplant diabetes mellitus patients and controls

Vascular capillary plexus	PTDM	Control	95% CI	P-value
Superficial				
Superficial central	19.36	21.86	-5.08 to 0.082	0.058
Superficial superior	46.63	46.91	-2.52 to 1.97	0.810
Superficial nasal	43.71	43,51	-1.55 to 1.94	0.825
Superficial inferior	46.22	46.4	-2.71 to 0.73	0.259
Superficial temporal	46.12	45.31	-0.55 to 2.17	0.244
Deep				
Deep central	18.61	20.66	-4.67 to 0.56	0.123
Deep superior	49.74	49.52	-2.17 to 2.62	0.857
Deep nasal	47.84	46.30	-0.89 to 3.97	0.214
Deep inferior	48.48	49.37	-3.10 to 1.32	0.430
Deep temporal	47.67	47.82	-1.76 to 1.46	0.855
Choriocapillaris				
Choriocapillaris central	37.45	37.70	-3.26 to 2.75	0.867
Choriocapillaris superior	51.83	51.54	-1.08 to 1.65	0.682
Choriocapillaris nasal	51.03	50.55	-0.79 to 1.74	0.466
Choriocapillaris inferior	51.07	51.84	-2.10 to 0.56	0.258
Choriocapillaris temporal	51.05	50.90	-0.89 to 1.17	0.787
FAZ				
FAZ deep plexus	449.93	388.61	-9.12 to 131.76	0.088

FAZ: foveal avascular zone

## Discussion

DR is the most common complication of diabetes and remains the leading cause of preventable blindness among working-age individuals in most developed countries [7]. PTDM is a specific and less common type of diabetes, and rather than being simply another form of type 2 diabetes, PTDM has its own pathophysiology [18]. The American Diabetes Association initially classified PTDM in the category of “other specific types” of diabetes. But at an expert meeting held in 2013 in Vienna, the terminology and consensus statement were updated [18, 19].

In addition to risk factors such as obesity and pre-transplant metabolic syndrome, post-transplant immunosuppression is a key risk factor for the development of PTDM. Widely used in immunosuppression, calcineurin inhibitors decrease insulin release from pancreatic beta cells and are associated with the development of PTDM [20–22]. Early corticosteroid withdrawal and cyclosporine use have shown to reduce the incidence of PTDM [23]. However, optimal immunosuppression to minimize the incidence of PTDM without increasing rejection rates and graft loss continues to be a subject of investigation.

Little is known about the long-term complications of PTDM, including DR which is the most common microvascular complication of types 1 and 2 DM. An analysis of United States Renal Data System (USRDS) data from the first 3 years after renal transplant showed an incidence of 8.3% of ophthalmic complications, but they were not specified [24]. A longitudinal study assessing general microvascular complications in renal transplant patients with PTDM found a lower-than-expected prevalence as well as a different clinical course of the complications, including no clinical findings of DR in color fundus images 5 years after diagnosis [10]. This single center study specifically addressed the OCT-detected retinal changes in renal transplant patients, with evidence of reduced nerve fiber and ganglion cell layers in patients with PTDM which seemed to signal probable preclinical diabetic retinopathy damage [10].

Spectral-domain OCT (SD-OCT) images have contributed to early findings in many diseases, such as reduced thickness of the inner retinal layers in patients with type 1 DM [25]. Technological advances such as OCTA have allowed further investigation of the progression of early morphological changes in the retina, and FAZ enlargement has been detected in patients with no or mild DR using OCTA [11, 13].

The purpose of the current study was to detect early signs of DR in neuroretinal layers and in the retinal microvasculature using OCTA in transplant recipients with diabetes, it complements and contributes to our previous findings in which we showed that PTDM decreased inner retinal thickness and without signs of clinical DR and now using OCT-A we suggest that this effect is mainly neurodegeration rather than vascular [10].

In our center, although no patient in the diabetic group showed clinical signs of DR even 5 years after diagnosis, there was a significant reduction in RNFL thickness and GCL + layers in transplant recipients who developed PTDM, which demonstrates early neuroretinal damage, but no significant decrease in microvascular density at all depth levels analyzed or changes in FAZ in the PTDM group, demonstrating that, at least at this time point, retinal microvasculature remained preserved.

The role of neurodegeneration in DR pathophysiology is becoming better understood over the years, and the concept of a primarily microvascular disease is now being discussed [26]. The term ‘neurovascular unit’ (NVU) was first applied to the central nervous system and its blood–brain barrier and refers to the functional network of neurons, glial cells, and highly specialized vasculature. In the retina, all the component cells of the NVU maintain the integrity of the inner blood–retinal barrier, and the impairment of the NVU is thought to be a primary event in DR pathogenesis [18, 19, 27].

Our findings contribute to the hypothesis that neurodegeneration might play a prior role in DR in transplant recipients with less evident early microvascular findings, and it may be a biomarker for subsequent retinal and vascular damage, but these data need to be confirmed by further evidence from longitudinal analysis. This study had limitations such as its cross-sectional design and its population consisting of patients that had undergone kidney transplant in a single center. Age-related thinning of neuroretinal parameters was controlled by adjusting the statistical model for age.

Longitudinal studies are warranted to elucidate the role of these early neurovascular changes in predicting the development of severe non-proliferative and proliferative DR. A better understanding of DR pathogenesis at the early stages of the disease is needed to contribute to new and more effective preventive strategies and treatments.

## Conclusions

The PTDM group had reduced inner neurosensory layers in the retina but no significant changes in vascular density according to OCTA, suggesting that early neuroretinal degeneration might occur prior to vascular changes secondary to PTDM. Prospective studies could help clarify the clinical course of retinal neuropathy and microvascular pathology in PTDM and provide a better understanding of PTDM complications.

### Electronic supplementary material

Below is the link to the electronic supplementary material.

Supplementary Material 1: Final text with Highlithed corrections.

Supplementary Material 2: Answer to the reviewers.

## Data Availability

The datasets analyzed during the current study are available from the corresponding author upon reasonable request.
