# Supplementary material for: Early retinal neurovascular findings in post-transplant diabetes mellitus patients without clinical signs of diabetic retinopathy
Source: Int J Retina Vitreous. 2023 Aug 23;9:49. doi: 10.1186/s40942-023-00487-4 (PMC10463975; doi:10.1186/s40942-023-00487-4)
Supplement: Supplementary file 1 — Supplementary Material 1: Final text with Highlithed corrections. [file 40942_2023_487_MOESM1_ESM.docx]

## **Early retinal neurovascular findings in post-transplant diabetes mellitus patients without clinical signs of diabetic retinopathy**

## Authors:

- Anne Elise Cruz do Carmo Chaves^1,5^
- Thizá Massaia Londero ^1,2^
- Monica Oliveira da Silva ^1^
- Fábio Lavinsky ^5^
- Cristiane Bauermann Leitão ^1,3^
- Andrea Carla Bauer ^1,4^
- Daniel Lavinsky ^1,5^

1. Post-Graduate Program in Medical Sciences, Endocrinology, Universidade Federal do Rio Grande do Sul (UFRGS), 97105-900 Porto Alegre, Rio Grande do Sul, Brazil
2. Clinical Medicine Department, Universidade Federal de Santa Maria, Santa Maria, Rio Grande do Sul, Brazil
3. Endocrinology Division, Hospital de Clínicas de Porto Alegre (HCPA), 90035-903 Porto Alegre, Rio Grande do Sul, Brazil;
4. Nephrology Division, Hospital de Clínicas de Porto Alegre (HCPA), 90035-903 Port Alegre, Rio Grande do Sul, Brazil;
5. Ophthalmology Division, Hospital de Clínicas de Porto Alegre, 90035-903 Porto Alegre, Rio Grande do Sul, Brazil

Corresponding author: Anne Elise Cruz do Carmo Chaves

## Email: [anneelisechaves@gmail.com](mailto:anneelisechaves@gmail.com)

## Mail: Av Soledade 250, 1102. Petrópolis Porto Alegre – RS, Brazil 90470540.

## Phone: +55 51 995663031

**Background:** Post-transplant diabetes mellitus (PTDM) is a specific subtype of diabetes with an uncertain impact on mortality and morbidity in post-transplant patients. Diabetic retinopathy is the most common microvascular complication of diabetes mellitus, but the long-term clinical progression in PTDM is unknown. New technologies are being used to assess pre-clinical signs of retinal changes, such as swept-source optical coherence tomography (OCT) and OCT-angiography. The aim of this study was to detect pre-clinical structural and vascular changes in the retina using swept-source-OCT and OCT-angiography in patients with PTDM. **Methods:** In this retrospective cohort study, post-kidney transplant patients were divided into PTDM and non-PTDM (control) groups. Both eyes of eligible PTDM patients and controls were included in this study. Inner retinal layer thickness was measured with swept-source-OCT. Retinal capillary density and the foveal avascular zone were measured with OCT-angiography. **Results:**  In the PTDM group, reduced thickness was found in the inferior ganglion cell layer plus inner plexiform layer (95% CI -8.76 to -0.68; p = 0.022) and the temporal inferior segment (95% CI -10.23 to -0.76; p = 0.024) of the inner retina, as well as in the retinal nerve fiber layer in the temporal (95% CI -34.78 to -9.28 p = 0.001) and temporal inferior segments (95% CI -33.26 to -5.03 p = 0.008). No significant differences were found in the vascular capillary plexus between groups at all depths, segments, or foveal avascular zone (p = 0.088). **Conclusions:** According to OCT-angiography, PTDM patients had reduced inner neurosensory retinal layers but no significant change in vascular density, which suggests that early neuroretinal degeneration might occur prior to vascular changes secondary to PTDM. Prospective studies could help elucidate the clinical course of retinal neuropathy and microvascular pathology in PTDM and provide a better understanding of PTDM complications.

Keywords: Post-transplant diabetes mellitus, diabetic retinopathy, early retinal neurovascular findings

## Introduction

Diabetes mellitus (DM) is a group of heterogeneous disorders involving increased blood glucose concentrations. Once hyperglycemia occurs, patients with all forms of diabetes are at risk of developing the same chronic complications, although the rates of progression may differ (1)(2). The International Diabetes Federation estimated the global population with diabetes mellitus to be 463,000,000 in 2019 and has projected it to be 700,000,000 by 2045 (3).

Post-transplant DM (PTDM), a subtype of diabetes first identified in 2014, occurs in previously nondiabetic people after a solid organ or hematopoietic transplantation (4). The pathogenesis of PTDM is related to the use of corticosteroids and calcineurin inhibitors in the post-transplant period and shares some of the same risk factors as type 2 DM, such as obesity and older age (5). PTDM is associated with higher rates of postoperative infection and cardiovascular events, which are the leading causes of death in kidney transplant recipients (6).

Diabetic retinopathy (DR) is the most common microvascular complication of DM and is the leading cause of vision loss in in the adult working population (7)(8). DR is typically classified according to a severity scale based on fundus examination findings (microaneurysms, hemorrhages, and vascular changes or neovascularization) (9). The long-term clinical progression in PTDM is unknown. Our group in a previous cohort of post-transplant patients with PTDM evaluate the clinical course of diabetic microvascular complications, assessing diabetic kidney disease (DKD), polyneuropathy, autonomic cardiovascular neuropathy as well as diabetic retinopathy. Was found no clinical signs of DR in retinography performed 5 years after diabetic diagnosis but at SS-OCT examination was observed difference between thickness of inner retinal layers in kidney transplant recipients with and without PTDM of more than 5 years. (10).

Many studies on DR have found pre-clinical abnormalities in retinal layer analyses using swept-source optical coherence tomography (SS-OCT) and optical coherence tomography angiography (OCTA) (11)(12)(13)(14)^.^ OCTA is a non-invasive imaging technique that generates volumetric angiography images in a few seconds. It provides a highly detailed reconstruction of the retinal vasculature, which allows for accurate delineation of the foveal avascular zone (FAZ) in diabetic eyes (15)(16) .

The aim of this study was to analyze specific retinal microvascular findings and detect early structural and vascular density changes in the retina, FAZ, and inner retinal layers using SS-OCT and OCTA in patients who have been diagnosed with PTDM for at least 5 years.

## Methods

A retrospective cohort study was conducted with all patients who received kidney transplant recipients at the Hospital de Clínicas de Porto Alegre (Porto Alegre, RS, Brazil) between January 2000 and December 2011. The Hospital’s ethics committee approved this study. All participants provided written informed consent, and this study complies with the principles of the Declaration of Helsinki and good clinical practice.

The inclusion criteria were at least 5 years since PTDM diagnosis, age > 18 years, and no history of DM prior to transplantation. PTDM was diagnosed according to the American Diabetes Association criteria,(17) and episodes of transient hyperglycemia related to high doses of corticosteroid and/or tacrolimus early after transplantation were not considered PTDM. The exclusion criteria were kidney graft loss, death, loss to follow-up, history of any significant ocular disease, previous diagnosis of glaucoma, or media opacities.

The control group consisted of kidney transplant patients during the same period with no diagnosis of PTDM. Exclusion criteria were kidney graft loss, death, or loss to follow-up, in addition to any ophthalmological condition that could interfere with the imaging assessment.

Both eyes of eligible PTDM patients and controls were included in this study. All patients underwent SS-OCT and OCTA with a DRI OCT Triton system (Topcon, Tokyo, Japan). Demographic data were also collected.

SS-OCT and OCTA Imaging

The images were analyzed in Topcon IMAGEnet 6 v1.22. The imaging protocol included wide macula, line macula, and simultaneous color fundus of the posterior pole. All patients were evaluated with SS-OCT by an experienced ophthalmologist, and all OCT scans were analyzed by a blinded investigator for quality, being excluded if they showed signs of low quality or a significant artifact.

OCTA scans were acquired using the DRI OCT Triton system based on the Topcon OCT angiography ratio analysis algorithm. The imaging protocol was a 6 x 6 mm Early Treatment DR Study grid overlay centered on the fovea. Boundary layer segmentations were defined as superficial capillary plexus from 2.6 μm below internal limiting membrane to 15.6 μm below the junction between inner plexiform layer and the inner nuclear layer, deep capillary plexus from 15.6 μm to 70.2 μm below this junction, and choriocapillaris from Bruch’s membrane (0-μm offset) to 10.4 μm below Bruch’s membrane. OCTA software determines the proportion of bright to dark pixels to derive a measure of vascular density. Vascular density is derived from the percentage of the area occupied by bright pixels in a segmented area. The FAZ (μm^2^) was measured through manual delineation in the deep plexus using SS-OCT software. Retinal thickness and vascular density measurements were obtained from an Early Treatment DR Study grid centered on the fovea (diameters: center 1 mm, inner circle 3 mm, outer circle 6 mm). Automated segmentation of the retina was performed in 9 Early Treatment DR Study areas, in addition to average thickness, center thickness, and total volume. The ganglion cell layer plus inner plexiform layer (GCL+), ie, from the nerve fiber layer/ganglion cell layer to the inner plexiform layer/inner nuclear layer, and the retinal nerve fiber layer (RNFL) segmentation, was determined in 6 standard areas: superior, nasal superior, temporal superior, inferior, temporal inferior, nasal inferior, and total volume.

Statistics

Demographic and descriptive data were expressed as mean (SD). Continuous variables were presented as mean  (SD), while categorical variables were presented as percentages. Differences between groups were assessed with generalized estimating equations and were adjusted for both eyes, excluding missing values. Statistical analysis was performed using IBM SPSS Statistics 28.0 (IBM, Armonk, NY, USA).

## Results

A total of 895 patients received a kidney transplant at the Hospital between January 2000 and December 2011, of whom 135 (15%) developed PTDM over 144.5 months of follow-up. Of these, 64 had been diagnosed with PTDM for > 5 years and were considered eligible for this study. After applying the exclusion criteria, 35 patients were included in PTDM group, and 48 patients without PTDM were included in control group. Most participants were male (n = 54, 65.06%), with a mean age was 53.78 years. The demographic and clinical findings are shown in Table 1.

The SS-OCT analysis consisted of 162 eyes (83 patients), 68 in the PTDM group and 94 in the control group. Age-adjusted inner retinal layer measurement showed reduced thickness in the PTDM GCL+ (95% CI -8.76 to -0.68; p = 0.022) and in the temporal inferior segment (95% CI -10.23 to -0.76; p = 0.024), as well as RNFL reduction in the temporal superior (95% CI -34.78 to -9.28; p = 0.001) and temporal inferior segments (95% CI -33.26 to -5.03; p = 0.008) (Table 2).

OCTA density was measured in 139 eyes, 61 in the PTDM group and 78 in the control group. After image quality assessment, 134 eyes remained eligible (73 participants): 58 eyes in PTDM group and 76 in control group. No significant difference was found between groups for the vascular capillary plexus at all depths and segments (superficial central density 95% CI -5.08 to 0.082; p = 0.058; deep central 95% CI -4.67 to 0.56; p = 0.123; choriocapillaris central 95% CI -3.26 to 2.75; p = 0.867). The mean capillary density values of the superficial capillary plexus, deep plexus, and choriocapillaris are shown in Table 3. No significant enlargement was observed in PTDM FAZ compared to controls (95% CI, -9.12 to 131.76; p = 0.088) (Table 3).

Table 1: Demographic and clinical characteristics of PTDM patients and non-PTDM controls

|  | **PTDM (35)** | **Controls (48)** | **p-value** |
| --- | --- | --- | --- |
| Age (years) | 58.31 | 51.04 | 0.001 |
| Sex – Male (%) | 57.14 | 70.83 | 0.495 |
| HbA1c (mean) | 7.54 | 5.42 | 0.001 |
| Caucasian (%) | 80 | 78.4 | 1.00 |
| Parental DM (%) | 40 | 43.2 | 0.931 |

DM: diabetes mellitus.

Table 2: Mean retinal layer thickness (μm) in PTDM patients and non-PTDM controls

| **Retinal Layer Thickness,**  **ETDRS (μm)** | **PTDM (μm)** | **Control (μm)** | **95%CI** | **p-value** |
| --- | --- | --- | --- | --- |
|  | **(n = 68 eyes)** | **(n = 94 eyes)** |  |  |
| Retina |  |  |  |  |
| Center retinal thickness | 188.16 | 193.26 | -19.72 to 9.54 | 0.495 |
| Total volume | 7.54 | 7.77 | -0.5 to 0.4 | 0.089 |
| Inner temporal | 285.06 | 294.65 | -22.54 to 3.38 | 0.147 |
| Inner superior | 295.86 | 304.80 | -21.28 to 3.41 | 0.156 |
| Inner nasal | 292.38 | 303.96 | -24.06 to 0.92 | 0.069 |
| Inner inferior | 293.55 | 302.71 | -21.59 to 3.28 | 0.149 |
| GCL+ |  |  |  |  |
| Total | 65.65 | 69.61 | -8.13 to 0.22 | 0.063 |
| Temporal superior | 66.49 | 69.18 | -7.34 to 1.96 | 0.257 |
| Superior | 64.06 | 67.57 | -7.87 to 0.86 | 0.115 |
| Nasal superior | 68.69 | 72.18 | -8.23 to 1.25 | 0.149 |
| Nasal inferior | 67.53 | 70.91 | -8.02 to 1.26 | 0.154 |
| Inferior | 61.64 | 66.36 | -8.76 to -0.68 | **0.022** |
| Temporal inferior | 65.45 | 71.25 | -10.23 to -0.76 | **0.024** |
| RNFL |  |  |  |  |
| Temporal | 70.12 | 74.85 | -11.51 to 2.04 | 0.171 |
| Temporal superior | 112.40 | 134.43 | -34.78 to -9.28 | **0.001** |
| Temporal inferior | 120.33 | 139.47 | -33.26 to -5.03 | **0.008** |
| Nasal superior | 111.98 | 117.35 | -18.02 to 7.27 | 0.406 |
| Nasal | 83.31 | 88.04 | -11.72 to 2.26 | 0.185 |
| Nasal inferior | 118.29 | 130.51 | -24.77 to 0.34 | 0.057 |

ETDRS: Early Treatment Diabetic Retinopathy Study; GCL+: Ganglion cell layer plus inner plexiform layer; RNFL: retinal nerve fiber layer

Table 3: Mean capillary density in optical coherence tomography angiography in post-transplant diabetes mellitus patients and controls

| Vascular capillary plexus | PTDM | Control | 95% CI | P-value |
| --- | --- | --- | --- | --- |
| Superficial |  |  |  |  |
| Superficial central | 19.36 | 21.86 | -5.08 to 0.082 | 0.058 |
| Superficial superior | 46.63 | 46.91 | -2.52 to 1.97 | 0.810 |
| Superficial nasal | 43.71 | 43,51 | -1.55 to 1.94 | 0.825 |
| Superficial inferior | 46.22 | 46.4 | -2.71 to 0.73 | 0.259 |
| Superficial temporal | 46.12 | 45.31 | -0.55 to 2.17 | 0.244 |
| Deep |  |  |  |  |
| Deep central | 18.61 | 20.66 | -4.67 to 0.56 | 0.123 |
| Deep superior | 49.74 | 49.52 | -2.17 to 2.62 | 0.857 |
| Deep nasal | 47.84 | 46.30 | -0.89 to 3.97 | 0.214 |
| Deep inferior | 48.48 | 49.37 | -3.10 to 1.32 | 0.430 |
| Deep temporal | 47.67 | 47.82 | -1.76 to 1.46 | 0.855 |
| Choriocapillaris |  |  |  |  |
| Choriocapillaris central | 37.45 | 37.70 | -3.26 to 2.75 | 0.867 |
| Choriocapillaris superior | 51.83 | 51.54 | -1.08 to 1.65 | 0.682 |
| Choriocapillaris nasal | 51.03 | 50.55 | -0.79 to 1.74 | 0.466 |
| Choriocapillaris inferior | 51.07 | 51.84 | -2.10 to 0.56 | 0.258 |
| Choriocapillaris temporal | 51.05 | 50.90 | -0.89 to 1.17 | 0.787 |
| FAZ |  |  |  |  |
| FAZ deep plexus | 449.93 | 388.61 | -9.12 to 131.76 | 0.088 |

FAZ: foveal avascular zone

Discussion

DR is the most common complication of diabetes and remains the leading cause of preventable blindness among working-age individuals in most developed countries (7). PTDM is a specific and less common type of diabetes, and rather than being simply another form of type 2 diabetes, PTDM has its own pathophysiology (18). The American Diabetes Association initially classified PTDM in the category of “other specific types” of diabetes. But at an expert meeting held in 2013 in Vienna, the terminology and consensus statement were updated (18)(19).

In addition to risk factors such as obesity and pre-transplant metabolic syndrome, post-transplant immunosuppression is a key risk factor for the development of PTDM. Widely used in immunosuppression, calcineurin inhibitors decrease insulin release from pancreatic beta cells and are associated with the development of PTDM (20–22). Early corticosteroid withdrawal and cyclosporine use have shown to reduce the incidence of PTDM (23). However, optimal immunosuppression to minimize the incidence of PTDM without increasing rejection rates and graft loss continues to be a subject of investigation.

Little is known about the long-term complications of PTDM, including DR which is the most common microvascular complication of types 1 and 2 DM. An analysis of United States Renal Data System (USRDS) data from the first 3 years after renal transplant showed an incidence of 8.3% of ophthalmic complications, but they were not specified (24). A longitudinal study assessing general microvascular complications in renal transplant patients with PTDM found a lower-than-expected prevalence as well as a different clinical course of the complications, including no clinical findings of DR in color fundus images 5 years after diagnosis (10). This single center study specifically addressed the OCT-detected retinal changes in renal transplant patients, with evidence of reduced nerve fiber and ganglion cell layers in patients with PTDM which seemed to signal probable preclinical diabetic retinopathy damage. (10).

Spectral-domain OCT (SD-OCT) images have contributed to early findings in many diseases, such as reduced thickness of the inner retinal layers in patients with type 1 DM (25). Technological advances such as OCTA have allowed further investigation of the progression of early morphological changes in the retina, and FAZ enlargement has been detected in patients with no or mild DR using OCTA (11)(13).

The purpose of the current study was to detect early signs of DR in neuroretinal layers and in the retinal microvasculature using OCTA in transplant recipients with diabetes, it complements and contributes to our previous findings in which we showed that PTDM decreased inner retinal thickness and without signs of clinical DR and now using OCT-A we suggest that this effect is mainly neurodegeration rather than vascular (10).

In our center, although no patient in the diabetic group showed clinical signs of DR even 5 years after diagnosis, there was a significant reduction in RNFL thickness and GCL+ layers in transplant recipients who developed PTDM, which demonstrates early neuroretinal damage, but no significant decrease in microvascular density at all depth levels analyzed or changes in FAZ in the PTDM group, demonstrating that, at least at this time point, retinal microvasculature remained preserved.

The role of neurodegeneration in DR pathophysiology is becoming better understood over the years, and the concept of a primarily microvascular disease is now being discussed (26). The term ‘neurovascular unit’ (NVU) was first applied to the central nervous system and its blood–brain barrier and refers to the functional network of neurons, glial cells, and highly specialized vasculature. In the retina, all the component cells of the NVU maintain the integrity of the inner blood–retinal barrier, and the impairment of the NVU is thought to be a primary event in DR pathogenesis (19)(18)(27)(28).

Our findings contribute to the hypothesis that neurodegeneration might play a prior role in DR in transplant recipients with less evident early microvascular findings, and it may be a biomarker for subsequent retinal and vascular damage, but these data need to be confirmed by further evidence from longitudinal analysis. This study had limitations such as its cross-sectional design and its population consisting of patients that had undergone kidney transplant in a single center. Age-related thinning of neuroretinal parameters was controlled by adjusting the statistical model for age.

Longitudinal studies are warranted to elucidate the role of these early neurovascular changes in predicting the development of severe non-proliferative and proliferative DR. A better understanding of DR pathogenesis at the early stages of the disease is needed to contribute to new and more effective preventive strategies and treatments.

Conclusions

The PTDM group had reduced inner neurosensory layers in the retina but no significant changes in vascular density according to OCTA, suggesting that early neuroretinal degeneration might occur prior to vascular changes secondary to PTDM. Prospective studies could help clarify the clinical course of retinal neuropathy and microvascular pathology in PTDM and provide a better understanding of PTDM complications.

References

1. Redmon JB, Olson LK, Armstrong MB, Greene MJ, Robertson RP. Effects of tacrolimus (FK506) on human insulin gene expression, insulin mRNA levels, and insulin secretion in HIT-T15 cells. J Clin Invest [Internet]. 1996 Dec 15;98(12):2786–93. Available from: https://doi.org/10.1172/JCI119105

2. Association AD. 2. Classification and Diagnosis of Diabetes: Standards of Medical Care in Diabetes—2018. Diabetes Care [Internet]. 2017 Nov 24;41(Supplement_1):S13–27. Available from: https://doi.org/10.2337/dc18-S002

3. Teo ZL, Tham Y-C, Yu M, Chee ML, Rim TH, Cheung N, et al. Global Prevalence of Diabetic Retinopathy and Projection of Burden through 2045: Systematic Review and Meta-analysis. Ophthalmology [Internet]. 2021 Nov 1;128(11):1580–91. Available from: https://doi.org/10.1016/j.ophtha.2021.04.027

4. Sharif A, Hecking M, de Vries APJ, Porrini E, Hornum M, Rasoul-Rockenschaub S, et al. Proceedings from an international consensus meeting on posttransplantation diabetes mellitus: recommendations and future directions. Vol. 14, American journal of transplantation : official journal of the American Society of Transplantation and the American Society of Transplant Surgeons. United States; 2014. p. 1992–2000.

5. Pinheiro Buarque MNA, de Francesco Daher E, de Matos Esmeraldo R, Lima Macedo RB, Martins Costa MC, Morais de Alencar CH, et al. Historical Cohort With Diabetes Mellitus After Kidney Transplantation and Associated Factors of Its Development in Adult Patients of a Transplantation Reference Center in the State of Ceará, Brazil. Transplant Proc [Internet]. 2014;46(6):1698–704. Available from: https://www.sciencedirect.com/science/article/pii/S0041134514003558

6. Yeh H, Lin C, Li Y-R, Yen C-L, Lee C-C, Chen J-S, et al. Temporal trends of incident diabetes mellitus and subsequent outcomes in patients receiving kidney transplantation: a national cohort study in Taiwan. Diabetol Metab Syndr [Internet]. 2020;12(1):34. Available from: https://doi.org/10.1186/s13098-020-00541-3

7. Yau JWY, Rogers SL, Kawasaki R, Lamoureux EL, Kowalski JW, Bek T, et al. Global Prevalence and Major Risk Factors of Diabetic Retinopathy. Diabetes Care [Internet]. 2012 Feb 10;35(3):556–64. Available from: https://doi.org/10.2337/dc11-1909

8. Wong TY, Cheung CMG, Larsen M, Sharma S, Simó R. Diabetic retinopathy. Nat Rev Dis Prim. 2016 Mar;2:16012.

9. Flaxel CJ, Adelman RA, Bailey ST, Fawzi A, Lim JI, Vemulakonda GA, et al. Diabetic retinopathy preferred practice pattern®. Ophthalmology. 2020;127(1):P66–145.

10. Londero TM, Giaretta LS, Farenzena LP, Manfro RC, Canani LH, Lavinsky D, et al. Microvascular Complications of Posttransplant Diabetes Mellitus in Kidney Transplant Recipients: A Longitudinal Study. J Clin Endocrinol Metab. 2018;104(2).

11. da Silva MO, do Carmo Chaves AEC, Gobbato GC, dos Reis MA, Lavinsky F, Schaan BD, et al. Early neurovascular retinal changes detected by swept-source OCT in type 2 diabetes and association with diabetic kidney disease. Int J Retin Vitr [Internet]. 2021;7(1):73. Available from: https://doi.org/10.1186/s40942-021-00347-z

12. Da Silva MO, Chaves AEC do C, Gobbato GC, Lavinsky F, Schaan BD, Lavinsky D. Early choroidal changes detected by swept-source OCT in type 2 diabetes and their association with diabetic kidney disease. BMJ open diabetes Res care. 2022 Nov;10(6).

13. Vujosevic S, Muraca A, Alkabes M, Villani E, Cavarzeran F, Rossetti L, et al. Early microvascular and neural changes in patients with type 1 and type 2 diabetes mellitus without clinical signs of diabetic retinopathy. Retina [Internet]. 2019;39(3). Available from: https://journals.lww.com/retinajournal/Fulltext/2019/03000/EARLY_MICROVASCULAR_AND_NEURAL_CHANGES_IN_PATIENTS.3.aspx

14. Lavinsky F, Lavinsky D. Novel perspectives on swept-source optical coherence tomography. Int J Retin Vitr. 2016;2:25.

15. de Carlo TE, Romano A, Waheed NK, Duker JS. A review of optical coherence tomography angiography (OCTA). Int J Retin Vitr [Internet]. 2015;1(1):5. Available from: https://doi.org/10.1186/s40942-015-0005-8

16. Chen Y-J, Khouri AS, Zarbin MA, Szirth BC. Early retinal microvascular abnormalities in young adults with type 1 diabetes mellitus without clinically evident diabetic retinopathy. Retina [Internet]. 2021;41(7). Available from: https://journals.lww.com/retinajournal/Fulltext/2021/07000/EARLY_RETINAL_MICROVASCULAR_ABNORMALITIES_IN_YOUNG.16.aspx

17. Association AD. Diagnosis and Classification of Diabetes Mellitus. Diabetes Care [Internet]. 2005 Jan 1;28(suppl_1):s37–42. Available from: https://doi.org/10.2337/diacare.28.suppl_1.S37

18. Davidson JA, Wilkinson A, Transplantation on behalf of the IEP on N-OD after. New-Onset Diabetes After Transplantation 2003 International Consensus Guidelines: An endocrinologist’s view. Diabetes Care [Internet]. 2004 Mar 1;27(3):805–12. Available from: https://doi.org/10.2337/diacare.27.3.805

19. Wilkinson A, Davidson J, Dotta F, Home PD, Keown P, Kiberd B, et al. Guidelines for the treatment and management of new-onset diabetes after transplantation. Clin Transplant. 2005 Jun;19(3):291–8.

20. Øzbay LA, Smidt K, Mortensen DM, Carstens J, Jørgensen KA, Rungby J. Cyclosporin and tacrolimus impair insulin secretion and transcriptional regulation in INS-1E beta-cells. Br J Pharmacol. 2011 Jan;162(1):136–46.

21. van Hooff JP, Christiaans MHL, van Duijnhoven EM. Evaluating mechanisms of post-transplant diabetes mellitus. Nephrol Dial Transplant Off Publ Eur Dial Transpl Assoc - Eur Ren Assoc. 2004 Dec;19 Suppl 6:vi8–12.

22. Heit JJ, Apelqvist AA, Gu X, Winslow MM, Neilson JR, Crabtree GR, et al. Calcineurin/NFAT signalling regulates pancreatic beta-cell growth and function. Nature. 2006 Sep;443(7109):345–9.

23. Pascual J, Royuela A, Galeano C, Crespo M, Zamora J. Very early steroid withdrawal or complete avoidance for kidney transplant recipients: a systematic review. Nephrol Dial Transplant Off Publ Eur Dial Transpl Assoc - Eur Ren Assoc. 2012 Feb;27(2):825–32.

24. Burroughs TE, Swindle J, Takemoto S, Lentine KL, Machnicki G, Irish WD, et al. Diabetic complications associated with new-onset diabetes mellitus in renal transplant recipients. Transplantation. 2007 Apr;83(8):1027–34.

25. van Dijk HW, Kok PHB, Garvin M, Sonka M, Devries JH, Michels RPJ, et al. Selective loss of inner retinal layer thickness in type 1 diabetic patients with minimal diabetic retinopathy. Invest Ophthalmol Vis Sci. 2009 Jul;50(7):3404–9.

26. Abcouwer SF, Gardner TW. Diabetic retinopathy: loss of neuroretinal adaptation to the diabetic metabolic environment. Ann N Y Acad Sci. 2014 Apr;1311:174–90.

27. Duh EJ, Sun JK, Stitt AW. Diabetic retinopathy: current understanding, mechanisms, and treatment strategies. JCI insight. 2017 Jul;2(14).

## Declarations

**Ethics approval and consent to participate:** The Hospital de Clínicas de Porto Alegre ethics committee approved this study. All participants provided written informed consent, and this study complies with the principles of the Declaration of Helsinki and good clinical practice.

**Consent for publication:** Not applicable

**Availability of data and materials:** The datasets analyzed during the current study are available from the corresponding author upon reasonable request.

**Competing interests:** The authors declare no competing interests.

**Funding:** This study received no external funding. It was conducted with the researchers’ own resources

**Author contributions:**

AECCC: Contributed to the study conception and to the acquisition, analysis, mand interpretation of the data, as well as to drafting the manuscript.

TML: Contributed to the study conception and data acquisition.

MOL: Contributed to the data analysis and interpretation.

CBL: Contributed to the study conception and data acquisition.

ACB: Contributed to the study conception and data acquisition.

DL: Contributed to the study conception, and to the acquisition, analysis, and interpretation of the data, as well as to revising the manuscript.
