# Supplementary material for: Early retinal neurovascular findings in post-transplant diabetes mellitus patients without clinical signs of diabetic retinopathy
Source: Int J Retina Vitreous. 2023 Aug 23;9:49. doi: 10.1186/s40942-023-00487-4 (PMC10463975; doi:10.1186/s40942-023-00487-4)
Supplement: Supplementary file 2 — Supplementary Material 2: Answer to the reviewers. [file 40942_2023_487_MOESM2_ESM.docx]

I would like to thank you for your comments regarding our article and answer the questions raised by the esteemed reviewers.

**Reviewer #1: The authors have carefully analyzed the retinal structure of patients with PTDM, an uncommon form of diabetes, and for which the ocular complications have received very little attention. Overall the paper is well-written and clear. Appropriate statistical methods to consider two-eye data were used. They found GCL+ and NFL thinning in the temporal and superior macula so the results seem likely to be valid. The lack of OCT-A density is interesting and unexpected, but their suggestion that this represents an early form of neurovascular impairment may be correct, particularly given that the mean HbA1c of the subjects was 7.54%.**

Thank you for your review and considerations about our paper. We believe that our data can contribute to understanding of diabetic retinopathy in PTDM, and the evidence of neurodegenaration before microvascular damage can be seen as a paradigm shift, not only in PTDM but perhaps in others diabetic retinopathy types.

**Reviewer #2: Its a good work .

You mentioned that about 760  patients were without PTDM , but only 48 patients were eligible in control group .
Could you please explain ?**

Thank you for your review and considerations about our paper. Our sample size has been defined based on the expected diabetic retinopathy prevalence of 6% in patients with type 1 diabetes after 4 years of diagnosis (Wisconsin Diabetes Registry Study) and considering the 64 patients with PTDM > 5 years in our cohort, we calculate a sample size (with correction for small populations) of 37 patients, plus 10% for losses and refusals, to detect diabetic retinopathy.

A sample size of 92 patients was calculated to detect an odds ratio of 3.3 between exposed (PTDM) and non-exposed (non-PTDM). We chose to use the largest sample size possible. Calculations were made through OpenEpi software (version 3.01, Emory University, Rollins School of Public Health), at 80% power and 95% confidence interval.

From a sample of 64 DMPT patients, 40 agreed to participate in the study. Some were excluded for corneal opacity, refusal, and image low quality. The final SS-OCT evaluation consisted in Eighty-three patients (35 DMPT e 48 NDMPT).

There was more than 48 eligible patients without PTDM, but we considered the number necessary to achieve our pre-established goal.
